# Rare purulent pericarditis caused by carbapenem-resistant *Acinetobacter baumannii*: A case report

**DOI:** 10.1097/MD.0000000000017034

**Published:** 2019-09-20

**Authors:** Jingjing Liu, Xuefei Xiao, Chaoqun Cen, Hong Yuan, Mingshi Yang

**Affiliations:** a Department of Intensive Medicine; b Department of Emergency; c Center of Clinical Pharmacology, Third Xiangya Hospital, Central South University, Changsha, Hunan, People's Republic of China.

**Keywords:** carbapenem-resistant *Acinetobacter baumannii*, purulent pericarditis

## Abstract

**Background:**

Pericardial infection caused by *Acinetobacter baumannii* is rare, particularly that of carbapenem-resistant *A baumannii* (CRAB).

**Case presentation:**

We describe a rare case of purulent pericarditis due to CRAB in a 76-year-old man with acute myocardial infarction and acute kidney injury. The man was admitted to the intensive care unit for a catheter-related bloodstream infection. Pericardial effusion was detected via the bedside X-ray and ultrasound, and pericardiocentesis was performed. Cultures of the pericardial fluid, catheter tip, and blood independently revealed the presence of CRAB. These findings confirmed a diagnosis of purulent pericarditis.

**Conclusions:**

Clinicians should be reminded that CRAB infection can lead to purulent pericarditis, particularly in patients with congestive heart failure or renal insufficiency.

## 1 Introduction

Purulent pericarditis is a rare disease.^[1]^ In the United States, from 2000 to 2011, the rate of pericarditis-related hospitalizations due to infections was 11 to 15 cases per 100,000 admissions.^[2]^ Purulent pericarditis is mostly diagnosed in immunocompromised individuals, and in adults with underlying diseases that affect the pericardial cavity.^[3,4]^ Mortality due to purulent pericarditis is as high as 100% in untreated patients, but effective treatment could lower the mortality of the patients.^[1]^ Thus, early diagnosis is of particular clinical importance for the prognosis of these patients.^[1]^

*Acinetobacter baumannii* (*A baumannii*) is a major cause of nosocomial infections. Because of extensive use of broad-spectrum antimicrobials during the last 2 decades, *A baumannii* has become increasingly resistant to drug therapies. Carbapenems are considered the treatment of choice for routine *A baumannii* infections, but carbapenem-resistant *A baumannii* (CRAB) is presently associated with significantly high rates of morbidity and mortality.^[5]^

Several bacterial agents are reported to cause purulent pericarditis, but pericardial infection caused by *A baumannii* is rare, with the last published case in an immunocompromised patient in 1997.^[6]^ Here we present an extremely unusual case of *A baumannii* pericarditis in an elderly patient.

## 2 Case presentation

A 76-year-old man was admitted for acute non-ST-segment elevated myocardial infarction, complicated with acute kidney injury. The medical history included hypertension. During hospitalization, continuous renal replacement therapy (CRRT) was performed for oliguria. After 2 weeks of hospitalization, the patient suffered from acute shiver and fever, and was transferred to the intensive care unit (ICU) due to acute dyspnea and low blood pressure.

**Figure 1:**
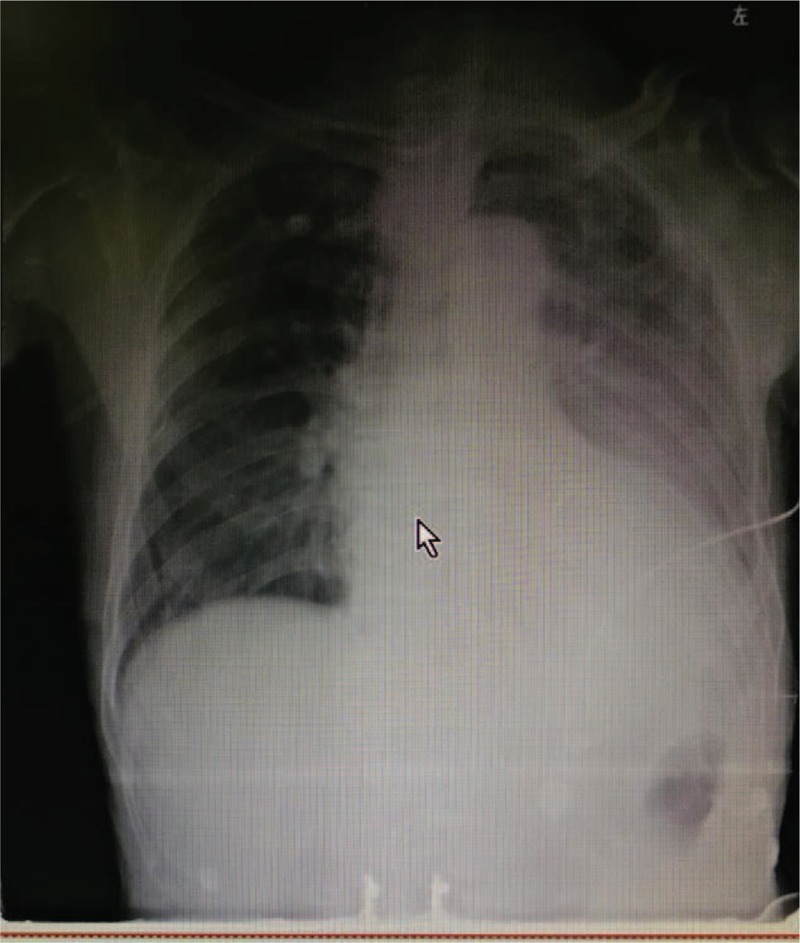
Chest X-ray at admission of ICU showed pulmonary infection with an enlarged heart. ICU = intensive care unit.

**Figure 2:**
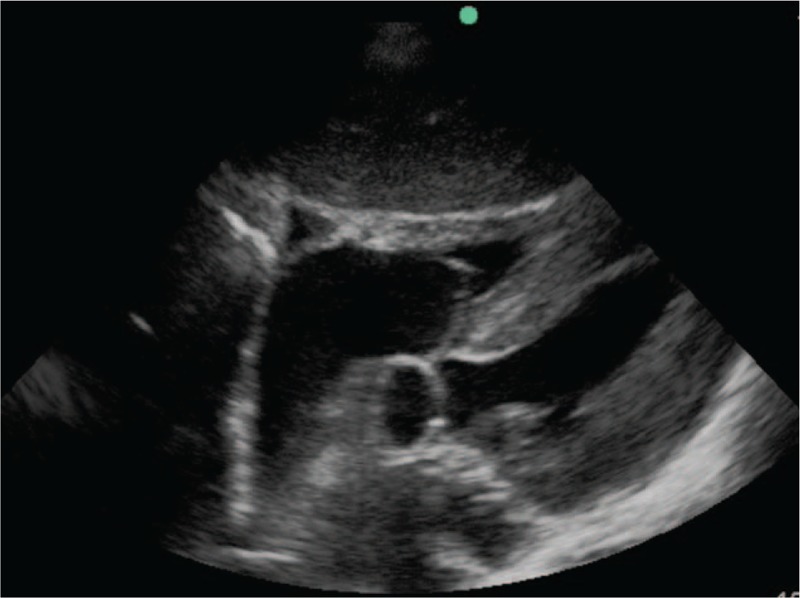
Echocardiographic examination at ICU admission showed an enlarged left atrium and left ventricle, and mild pericardial effusion. ICU = intensive care unit.

Upon ICU admission, the physical examination showed a temperature of 38.5°C, heart rate 108 beats/minute, respiration 30 breathes/minutes, blood pressure 101/57 mm Hg with intravenous norepinephrine, and 88% percutaneous saturation of oxygen. The patient appeared to be confused. Auscultation of the lungs revealed obvious dry and wet rales, and the border of the heart expanded toward the left inferior. The heart rate was regular, the abdomen was flat without tenderness, and both legs were without edema. The blood showed white blood cells at 10.4 × 10^9^ (neutrophil cells, 87.5%), hemoglobin 97 g/L, and platelet count 41 × 10^9^. Blood gas analyses showed pH 7.27, partial pressure of carbon dioxide 21.8 mm Hg, partial pressure of oxygen 57.8 mm Hg, base deficit −15.8 mmol/L, bicarbonate 12.2 mmol/L, and lactate 4.4 mmol/L. Blood urea nitrogen was 18.83 mmol/L, serum creatinine was 735 μmol/L, and procalcitonin was 142.56 ng/mL. The chest X-ray showed pulmonary infection with an enlarged heart (Fig. 1). The echocardiographic examination showed an enlarged left atrium and left ventricle, and mild pericardial effusion (Fig. 2).

In the ICU, the patient received tracheal intubation with mechanical ventilation. Intravenous norepinephrine was continuously administered to maintain the blood pressure. Meropenem (0.5, i.v. q8 h) was initially administered as an experience-guided anti-infection treatment. After 3 days, the temperature of the patient went down significantly, fluctuating between 37.2°C and 37.8°C, and the dose of intravenous norepinephrine was reduced. However, the platelet blood count continued to decrease. On the fourth day in the ICU, results of culture of the CRRT catheter and blood culture both showed CRAB.

**Figure 3:**
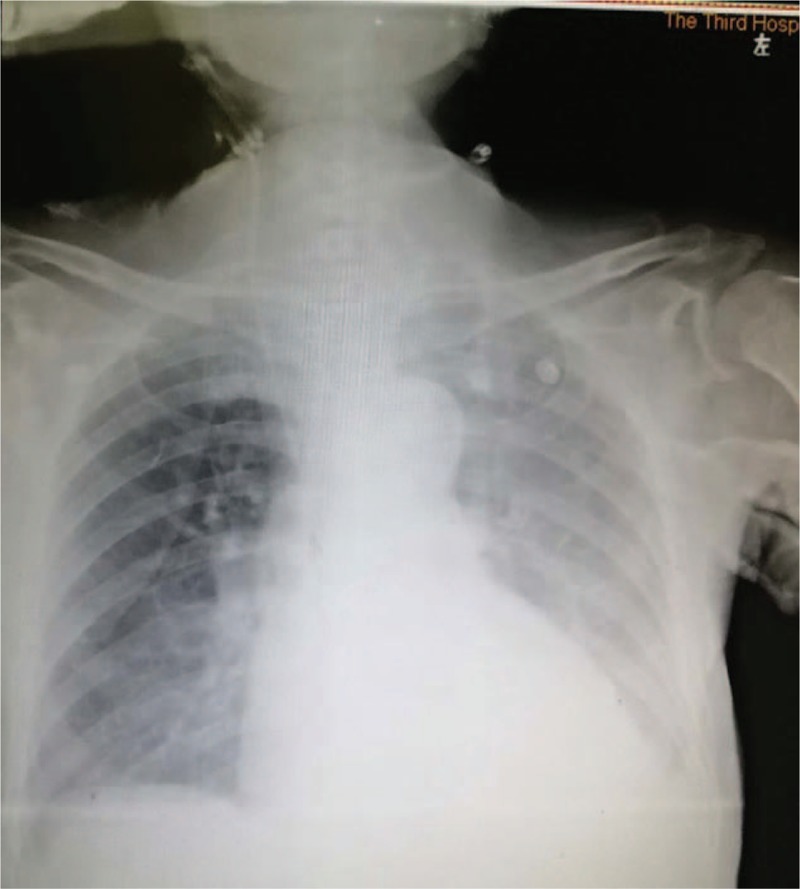
Bedside chest X-ray showed an enlarged heart.

**Figure 4:**
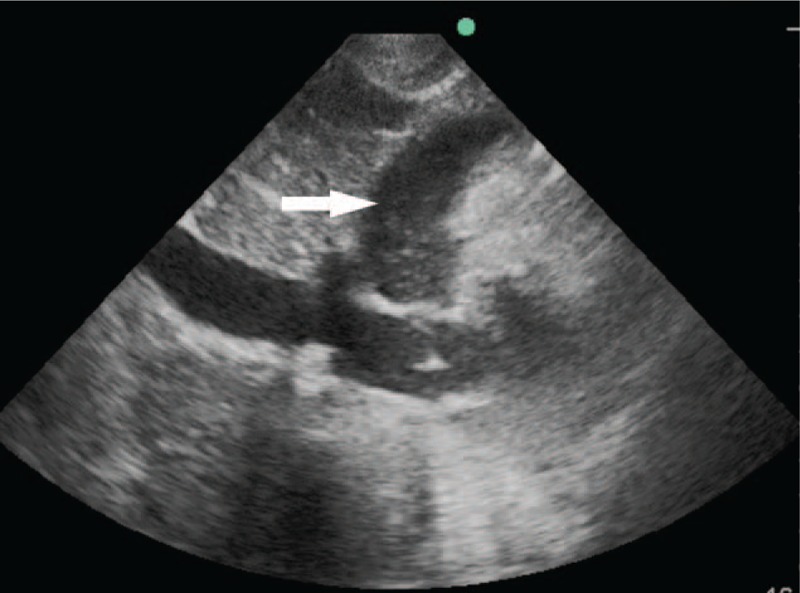
Bedside echocardiography revealed pericardial effusion.

**Figure 5:**
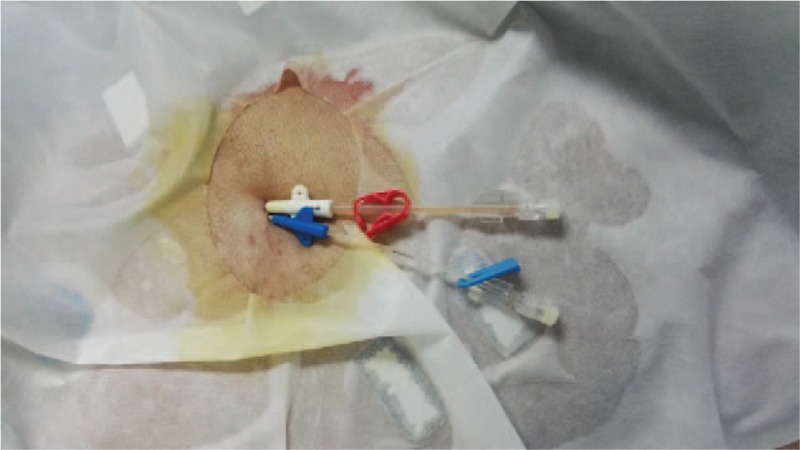
Purulent fluid from the pericardial effusion was obtained by pericardiocentesis in the ICU.

**Figure 6:**
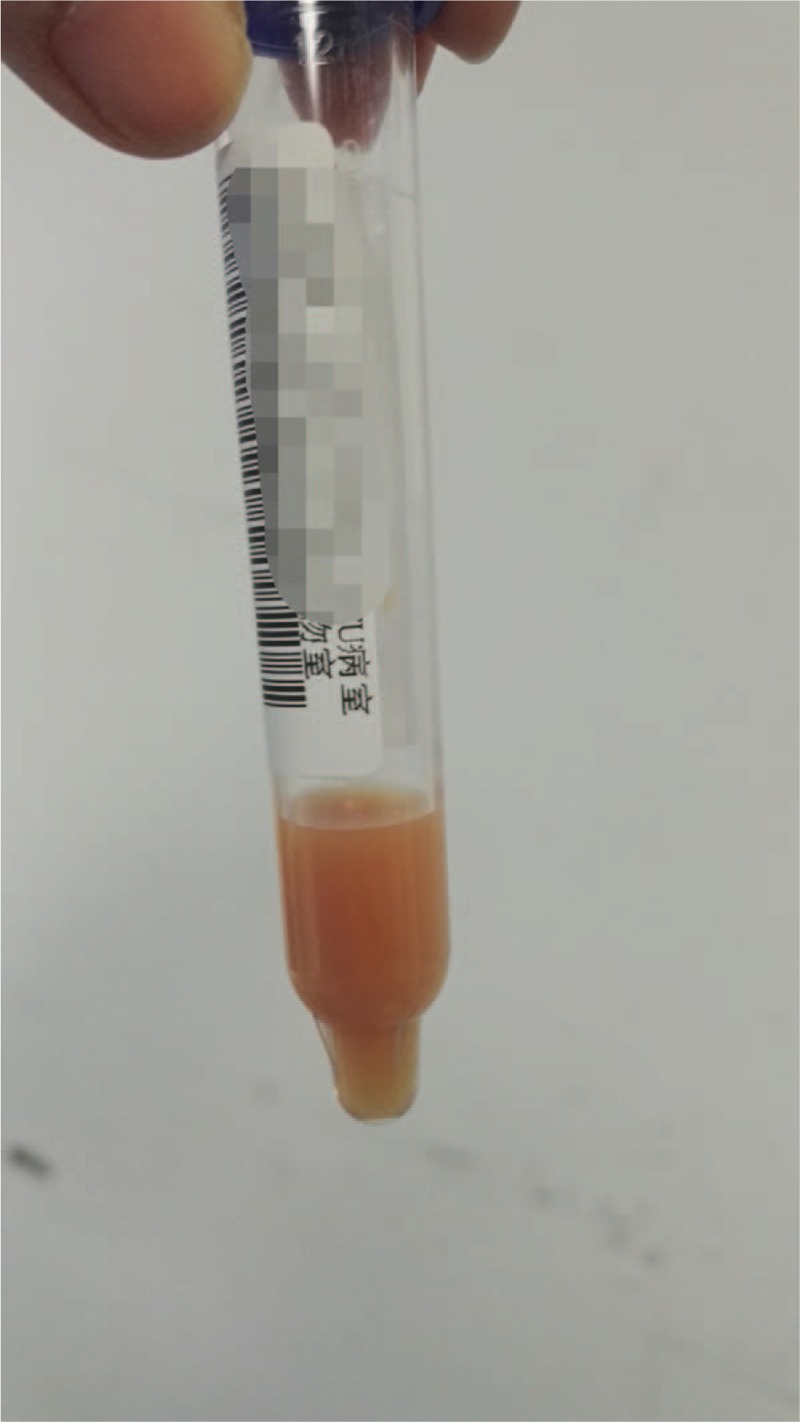
Pericardiocentesis retrieved purulent fluid.

Based upon the above signs, the diagnosis was made of catheter-related bloodstream infection, septic shock, and multiorgan failure. Based on drug sensitivity analyses, the antibiotics were adjusted to tigecycline combined with sulbactam according to the results of antibiotic sensitive test (moderate sensitivity, Clinical and Laboratory Standards Institute standard). At 1 week after ICU admission, the patient suffered an acute decrease in blood pressure, and auscultation revealed the sound of a weak heart. Bedside chest X-ray showed an enlarged heart (Fig. 3), and the bedside echocardiographic examination indicated pericardial effusion (Figs. 4 and 5). Pericardiocentesis was performed immediately, and purulent fluid from the pericardial effusion was obtained (Fig. 6). Culture of the pericardial fluid confirmed CRAB infection. The patient died of sepsis-related multiple organ failure eventually.

## 3 Discussion

*A baumannii* is an aerobic, pleomorphic, and nonmotile gram-negative coccobacillus that has a high incidence rate among hospitalized patients.^[7]^*A baumannii* has been implicated in a wide spectrum of infections, including ventilator-associated pneumonia, meningitis, and infections of the bloodstream, skin and soft-tissues, urinary tract, and surgical sites.^[8]^ The pathogen has a particular ability to develop rapid resistance to multiple antimicrobial agents, occasionally including carbapenem and polymyxin, making it a serious threat to inpatients. Rates of carbapenem resistance in the United States have increased from 9% in 1995 to 40% in 2004, and in Taiwan from 14% in 2003 to 46% in 2008.^[9,10]^

CRAB is associated with extremely high mortality in hospitalized patients.^[11]^ Bloodstream infection is particularly important, with an even higher mortality rate (29%–59%).^[12,13]^ Independent risk factors of multidrug-resistant *A baumannii* bacteremia are older age, pneumonia, use of drainage catheters, and ICU stay.^[13]^ The pericardium is a rare site, for which the etiology is bloodstream infection. To date, only a few cases have been reported, most of which were caused by gram-positive bacteria such as *Staphylococcus aureus* and *Streptococcus pneumoniae*.^[14,15]^

In the current era of antibiotic resistance, gram-negative enteric bacteria are a frequent cause of bacterial pericarditis.^[4,16]^ Although *A baumannii* is frequently isolated in the ICU, it is seldom identified from the pericardial cavity. An early case report in 1997 identified *A baumannii*-related pericarditis in an immunocompromised patient with systemic lupus erythematosus.^[6]^ A recent case series from Iran also identified *A baumannii* as a rare pathogen in pericarditis (1 in 35 patients; 3%).^[17]^ However, to the best of our knowledge, this is the first report of severe purulent pericarditis caused by CRAB.

For patients with severe congestive heart failure or renal dysfunction, pericardial effusion is not rare. However, in most cases, pericardial effusion tends to be exudate or transudate, with no bacterial infection. Pleural effusion is also common in these patients. Since fluid restriction and diuresis are effective for these patients, pericardiocentesis may not be routinely performed. This is the reason that pericardiocentesis was not initially performed in the present case, when pericardial effusion was detected upon admission to the ICU.

Based on our findings, clinicians should be reminded that CRAB infection can lead to purulent pericarditis. Particularly in patients with congestive heart failure or renal insufficiency, regular and continuous monitoring of the pericardial effusion with bedside echocardiography is needed. If the peripheral effusion becomes acutely severe with no particular inducing factors (such as fluid overload or insufficient dialysis) diagnostic pericardiocentesis should be performed immediately.

## Author contributions

**Conceptualization:** Xuefei Xiao, Hong Yuan.

**Data curation:** Jingjing Liu, Xuefei Xiao, Chaoqun Cen.

**Funding acquisition:** Mingshi Yang.

**Methodology:** Chaoqun Cen.

**Project administration:** Xuefei Xiao.

**Supervision:** Mingshi Yang.

**Writing – original draft:** Jingjing Liu.

**Writing – review & editing:** Jingjing Liu, Hong Yuan.
